# Do Fibromyalgia Patients Have a Preferred Resistance Training Volume? Protocol for a Randomized Crossover Clinical Trial

**DOI:** 10.1002/pri.70101

**Published:** 2025-08-31

**Authors:** Bruno Honório Cavalcanti, André Pontes‐Silva, Clécio Gabriel de Souza, Mariana Arias Avila, Marcelo Cardoso de Souza

**Affiliations:** ^1^ Physical Therapy Department Universidade Federal do Rio Grande do Norte Natal Brazil; ^2^ Study Group on Chronic Pain (NEDoC) Laboratory of Research on Electrophysical Agents (LAREF) Postgraduate Program in Physical Therapy Department of Physical Therapy Universidade Federal de São Carlos (UFSCar) São Carlos Brazil

**Keywords:** exercise, fibromyalgia, pain, resistance training

## Abstract

**Background:**

Do fibromyalgia patients have a preferred resistance training volume?

**Purpose:**

This protocol will support a trial evaluating the preference for volume in three different resistance training protocols for women with fibromyalgia.

**Methods:**

Protocol for a randomized crossover clinical trial. Women aged 18–65 years with a diagnosis of fibromyalgia will be included. Patients who have undergone resistance training in the last 6 months, who have another associated rheumatic disease, who have travel or scheduling commitments that will require them to be absent for the next 4 (four) weeks from the start of the study, who have upper and/or lower extremity musculoskeletal injuries, or who have cardiac problems that prevent maximal and submaximal exercise will not be included. The primary outcome will be preference. Secondary outcomes will be expectation, pain intensity, affect, and perceived effort, training volume, and cadence. For the primary outcome, the number of choices of the 3 types of training will be recorded as a percentage. For the analysis of the primary outcome, we will summarize the patient's preference in a contingency table, compare the proportions using the chi‐squared test, and finally verify the effect size of the observed differences. For the secondary outcomes, statistical analyses will be performed by a blind statistician. The independent variables of group and time will be considered for each dependent variable.

**Results:**

This will be the first study to examine the preference of women with fibromyalgia for different volumes of resistance training.

**Discussion:**

We will show whether exercise volume preference is related to subjective perception of exertion and pain intensity in women with fibromyalgia.

**Trial Registration:**

ClinicalTrials.gov (Identifier: NCT06424743)

## 1 Introduction

Fibromyalgia is a painful syndrome of unknown etiology that affects at least 2% of the world's population (Häuser et al. 2015). Although pain is a major symptom, other symptoms such as non‐restorative sleep, fatigue, and mood disturbances are common (Pontes‐Silva, Nunes, et al. 2023). The benefits of resistance training for patients with fibromyalgia have been well established in the literature and include: improvement in pain, function, quality of life, pain points and physical conditioning (Häuser et al. 2015).

Recent evidence underscores the intricacy of fibromyalgia's pathophysiology. This includes dysfunctions in central nervous system pain modulation, neuroinflammation, and altered hypothalamic–pituitary–adrenal axis activity. These factors collectively help to explain the persistence and multisystemic nature of symptoms. These findings underscore the importance of non‐pharmacological approaches that address not only symptoms but also the broader biopsychosocial context of the disease. In this regard, physical exercise, including resistance training, is a key therapeutic strategy due to its potential neurophysiological benefits beyond symptom relief (González‐Álvarez, Riquelme‐Aguado, Rossettini, et al. 2024).

Although resistance training is currently part of the accepted non‐pharmacological treatment for fibromyalgia, we do not have concrete guidelines regarding its prescription in terms of acute training variables such as intensity, order of exercises, speed of execution, type of exercise, and training volume (Macfarlane et al. 2017). Assuming the importance of the acute variables of resistance training, which have already been recognized and studied, it is reasonable to accept that the training volume is equivalent to the dose of a medication, where the variation of this prescribed amount will produce different results in individuals (Schoenfeld et al. 2017).

From an exercise science perspective, training volume—typically defined as the total amount of work performed (sets × repetitions × load)—has been consistently associated with the magnitude of physiological adaptations. This finding reinforces the idea that training volume is a primary determinant of efficacy (Schoenfeld et al. 2017). Another study observed significant benefits with varying volumes of resistance training in clinical populations such as those with fibromyalgia (Forner‐Álvarez et al. 2024).

This suggests that volume modulation can be viewed similarly to medication dosage, where specific amounts yield different therapeutic effects. This analogy supports the idea that prescribing volume, rather than intensity, plays a central role in optimizing the safety and efficacy of resistance training for patients with fibromyalgia (Schoenfeld et al. 2017; Forner‐Álvarez et al. 2024).

Muscle dysfunction, including mitochondrial alterations and reduced oxidative capacity, has been reported in patients with fibromyalgia. This dysfunction contributes to fatigue, pain, and exercise intolerance. These impairments underscore the importance of resistance training as a treatment for these symptoms and as a means of improving muscle health and energy metabolism in these individuals. It is crucial to understand this muscular profile when tailoring resistance training programs to avoid overexertion and enhance adherence (González‐Álvarez, Riquelme‐Aguado, González‐Pérez, et al. 2024).

Given the high dropout rate and the belief among fibromyalgia patients that exercise worsens their symptoms, an incorrect dosage may cause the patient to abandon the exercise program, in addition to creating a negative exercise experience (Vancampfort et al. 2024). The current literature still does not provide clear guidance on what exercise volume can be used for fibromyalgia patients. This gap presents a barrier for clinicians prescribing and supervising resistance training for this type of population (Pontes‐Silva, Dibai‐Filho, et al. 2023).

Although the benefits of exercise are evident, there is wide variability in how individuals respond, and many patients still struggle to adhere to an exercise regimen. A recent systematic review revealed that physical and psychological barriers, such as pain during or after exercise, fear of worsening symptoms, and lack of motivation, significantly impact exercise adherence in patients with fibromyalgia. This underscores the importance of considering not only physiological outcomes but also patient experience and engagement with the training process (Viceconti et al. 2022).

In addition, some studies that have demonstrated the efficacy of resistance training have found that even when we find an effective dose for treating fibromyalgia patients, this appropriate dose does not guarantee that the patient will adhere to regular resistance training (Rausch Osthoff et al. 2018). In general, the dose of resistance training meets only two implications: the positive result of this prescribed dose (the result, i.e., whether this dose improves the symptom, in the case of fibromyalgia) and the safety (absence of injury or a very low percentage) of this dose (Ekkekakis et al. 2011).

In contrast, when exercise prescription is effective and safe, the results themselves do not guarantee reproducibility for other contexts and populations, depending exclusively on the patient's will (i.e., the specificity of each individual). Among the many individual specificities, we highlight patient preference, which has been recommended for over 2 decades, as it is one of the pillars of evidence‐based practice (Garber et al. 2011). In addition, patient preference is a specific recommendation for fibromyalgia (Macfarlane et al. 2017; Rausch Osthoff et al. 2018).

Patient‐centered approaches, particularly those that consider individual preferences, have become increasingly important in fibromyalgia management. Recently, experts have recommended personalized exercise interventions based on patient goals, symptoms, and preferences to optimize long‐term adherence and clinical outcomes. These recommendations align with evidence‐based practices and justify exploring how specific variables, such as training volume, align with patient preferences (Viceconti et al. 2021).

The literature has already demonstrated positive results in relation to the most varied training volumes in patients with fibromyalgia, and how to plan an intervention program; however, there are no studies with more specific investigations in relation to the volume of resistance training and the preference of patients with fibromyalgia (Pontes‐Silva, Dibai‐Filho, et al. 2023). Thus, under the scientific hypothesis that women with fibromyalgia prefer lower volumes of resistance training, this protocol will support a trial evaluating the preference for volume in three different resistance training protocols for women with fibromyalgia.

## 2 Methods

### 2.1 Trial Design and Setting and Ethics Aspects

The protocol for a randomized crossover clinical trial (ClinicalTrials.gov: NCT06424743) was approved by the Research Ethics Committee of the Universidade Federal do Rio Grande do Norte (report number: 6.303.419), Brazil. The SPIRIT 2025 Statement was used (Hróbjartsson et al. 2025). The trial will be reported according to the Consolidated Standards of Reporting Trials and the exercise program according to the Template for Intervention Description and Replication (Schulz et al. 2010; Hoffmann et al. 2014).

### 2.2 Patient, Public Involvement, and Recruitment

**FIGURE 1:**
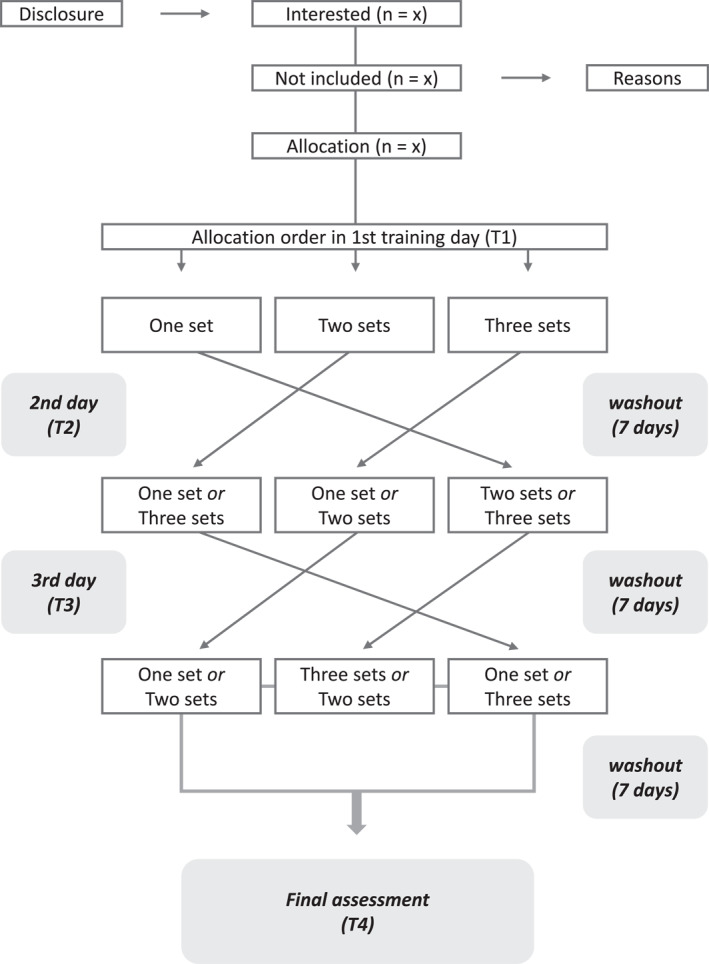
Flowchart. T1: Assessment will be performed respecting a 7‐day interval after the baseline assessment. T2 and T3: assessments will follow the same procedure as T1, with a 7‐day interval between each protocol (T1, T2 and T3), so that the patient will perform the other two training protocols. T4: assessment one week after the T3 assessment.

Patients will be recruited through social media promotion of the research. Initial contact will be made by Researcher A by telephone to schedule the screening. If eligible, patients will be informed of the objectives and procedures of the study and will sign an informed consent form without any influence from the researcher. Sociodemographic and fibromyalgia impact data will be collected. Patients' personal data will be numerically coded and stored in a database to maintain blinding of the study (Figure 1).

### 2.3 Eligibility Criteria

Women aged 18–65 years with a diagnosis of fibromyalgia according to the 2016 American College of Rheumatology criteria will be included (Wolfe et al. 2016). Patients who have undergone resistance training in the last 6 months, who have another associated rheumatic disease, who have travel or scheduling commitments that will require them to be absent for the next 4 (four) weeks from the start of the study, who have upper and/or lower extremity musculoskeletal injuries, or who have cardiac problems that prevent maximal and submaximal exercise will not be included.

### 2.4 Harms and Research Team

This randomized crossover clinical trial will involve five researchers who will perform the following functions: (A) screening and blinded assessment of patients; (B) randomization of patients; (C) monitoring of training protocols; (D) tabulation of data collected during assessments; and (E) statistical analysis.

### 2.5 Randomization, Allocation, Blinding, Patient Timeline, and Data Collection Methods

Randomization will be performed before each training protocol, taking into account the 3 different types of training protocols. The order in which the patient will perform the training protocol will be determined by a simple draw in an envelope containing 3 papers described as 1 series, 2 series, or 3 series. Therefore, if in the first session the patient draws the paper described as 1 series, in the following week this paper will be removed from the envelope and the same will draw 2 or 3 series, and so on.

In this way, all patients involved in the study will randomly perform the 3 types of protocols proposed in the study, and they will be their own controls. To avoid allocation bias, opaque sealed envelopes will be used, with sequential numbers on the outside of the envelope. This allocation will be made by Researcher B, who will not be involved in any other study procedures. Patients selected in the screening conducted by Researcher A who agree to participate in the research will be randomly assigned.

Regarding blinding, all assessments will be performed by Researcher A, who will not be involved in the interventions and will not participate in the delivery of the training protocol. Each patient will remain identified by numbers and not by name. In order not to compromise the blinding of the study, all patients will undergo the same assessment before and after the training session. Due to the nature of the proposed intervention (physical exercise), the therapist and patients will not be blinded to the intervention. Patients will be instructed not to disclose any information to other patients and/or researchers.

**FIGURE 2:**
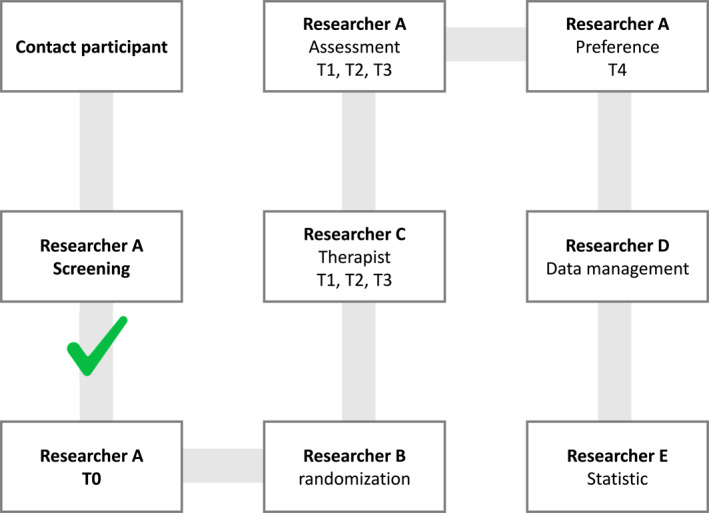
Research team and flow. T0: Initial assessment (baseline). T1: Assessment will be performed respecting a 7‐day interval after the baseline assessment. T2 and T3: Assessment will follow the same procedure as T1, with a 7‐day interval between each protocol (T1, T2 and T3), so that the patient will perform the other two training protocols. T4: assessment one week after the T3 assessment.

At the end of the T3 training protocol, with a 7‐day interval between these assessments, we will conduct T4, where each patient will be asked which training protocol they prefer. After T4, researcher D will tabulate the data, which will be delivered to researcher E, who will be responsible for the statistical analysis and who will not participate in the previous phases of the research (Figure 2).

### 2.6 Intervention and Comparator

**FIGURE 3:**
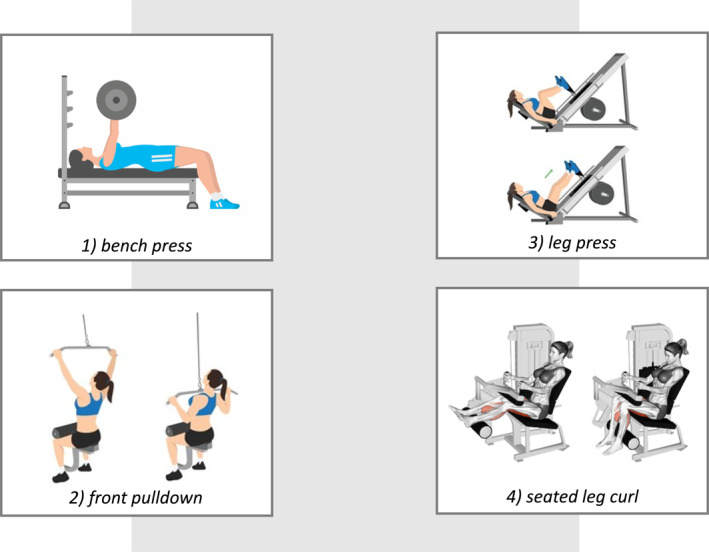
Proposed exercises.

The research will be conducted in a *blinded* manner after the publication of this protocol. Before the start of the study, a series of training steps will be implemented to carry out the assessments and training programs. The initial assessment T0 consists of explaining how the study will be conducted, the importance of the research, collecting anthropometric variables (body mass, stature, and body mass index), assessing clinical aspects and the impact that fibromyalgia causes to the patient and other variables such as pain intensity and affect. In addition, familiarization with the exercises and testing of maximum repetition loads will be performed in the following exercises: bench press, front pulldown, leg press, and seated leg curl. The loads found in this evaluation are used in the training protocols (Figure 3).

The T1 assessment will be performed respecting a 7‐day interval after the T0 assessment. At this interval, the patient will return and we will perform the first training protocol drawn on that day (T1). In addition, we will collect the following variables: pain intensity and affect before and after training, total training volume, and subjective effort after training.

The T2 and T3 assessments will follow the same procedure as T1, with a 7‐day interval between each protocol (T1, T2 and T3), so that the patient will perform the other two training protocols. The protocol draw will take place on the respective days of each assessment (T2) and (T3). If the envelope of the protocol drawn has already been performed on previous days, the patient will be instructed to perform a new draw.

Finally, at the T4 assessment, one week after the T3 assessment, the patient will be asked by telephone by the same evaluator (T0 baseline) about his preference for the programs performed in the study (with 1, 2 or 3 sets), standardizing the same commands used in person throughout the study. The following is a description of the three types of training programs with different volumes. The only different training variable will be the number of sets in the three training protocols of the study (Table 1).

**TABLE 1:** Training protocol.

Training (protocol)	Exercise	Sets (*n*)	Repetitions (*n*)	Load (1RM)	Rest (minute)
1	1–4	1	10	60%	2
2		2			
3		3			

*Note:* 1RM: One‐repetition maximum test. Each patient will receive the exercise session individually, as each of the three training protocols has different volumes (i.e., 1, 2, or 3 sets), consequently, the durations will vary for each patient; and then the durations will be compared between the groups.

### 2.7 Assessments and Data Management

In order to meet the research objectives, the initial assessments will be detailed to establish the baseline of the study, in addition to the examination data of the predetermined outcomes. Information such as: age, level of education, anthropometric data (stature and body mass), time of diagnosis, weekly physical activity time, patient expectations regarding the proposed training protocols and clinical data of the patient, such as the impact caused by the disease. These data will only be collected during the initial assessment in order to characterize the sample of patients.

### 2.8 Load Test

To determine the training loads that will be used in this research, we chose to use a regression equation that predicts the value of the maximum load by performing a submaximal effort in the exercises proposed by the research, for the following reasons: minimizing the risk of injury, for better clinical applicability, and for the high degree of reliability compared to the one‐repetition maximum test in resistance exercises (O'Connor and Simmons 1989).

In addition, the study will evaluate women with fibromyalgia who have low exercise tolerance and high activity withdrawal (Vancampfort et al. 2024). To this end, we selected a submaximal effort test with the best reliability and reproducibility compared to the widely used one‐repetition maximum test, and thus found a prediction equation with the highest correlation and lowest standard error correlated with the one‐repetition maximum test, namely, one repetition maximum = load × [1 + (0.025 × lreps)] (Lacio et al. 2010).

The warm‐up program will be the same as that used in the conventional one‐repetition maximum test, where: each patient will perform a series of 5–10 repetitions, with a load equivalent to 40%–60% of the maximum load perceived before each proposed exercise. After respecting a 2‐min recovery interval after warming up, the subject will perform a series with the highest number of repetitions he/she can in each exercise. At this time, the evaluator will instruct the patient as follows: “Now, perform the highest number of repetitions you can”.

The evaluation of the loads in the exercises proposed in this research will be carried out in the T0 evaluation. The exercises, barbell bench press, front pulldown, 45° leg press, and seated leg curl, will be performed on supertech machines.

### 2.9 Outcomes

The primary outcome will be preference. Preference will be assessed by the following question: “After completing the training sessions, which one did you prefer?”. The preference will be assessed at T5. Secondary outcomes will be expectation, pain intensity, affect, and perceived effort, training volume, and cadence. Table 2 summarizes the assessment times and their respective outcomes.

**TABLE 2:** Outcome assessments.

Variables	T0	T1			T2			T3			T4
		A	B	C	A	B	C	A	B	C	
Primary outcome											
Preference											✓
Secondary outcome											
Expectation	✓										
Pain intensity	✓	✓	✓	✓	✓	✓	✓	✓	✓	✓	
Affect		✓	✓	✓	✓	✓	✓	✓	✓	✓	
Perceived effort			✓			✓			✓		
Training volume			✓			✓			✓		
Cadence			✓			✓			✓		

*Note:* A: Pre. B: Post: C: 24h. T0: Initial assessment. T1: Assessment will be performed respecting a 7‐day interval after the T0 assessment. T2 and T3: assessments will follow the same procedure as T1, with a 7‐day interval between each protocol (T1, T2 and T3), so that the patient will perform the other two training protocols (Figures 1, 2, 3). T4: assessment one week after the T3 assessment.

### 2.10 Expectation

The Likert scale will be used for treatment expectations. This scale is designed to assess the patient's expectations of the treatment they will receive at the beginning of the study with the following questions (Beasley et al. 2017):

- Do you think that if you start the workout with 1 set, you will: (1) get a lot worse, (2) get a little worse, (3) neither get better nor worse, (4) get a little better, and (5) get a lot better.
- You think that if you start the workout with 2 sets, you will: (1) get a lot worse, (2) get a little worse, (3) neither get better nor worse, (4) get a little better, and (5) get a lot better.
- You think that if you start the workout with 3 sets, you will: (1) get a lot worse, (2) get a little worse, (3) neither get better nor worse, (4) get a little better, and (5) get a lot better.

### 2.11 Pain Intensity

The intensity of widespread pain is assessed using the numerical pain rating scale, a self‐report instrument validated for Portuguese (Ferreira‐Valente et al. 2011). The Numeric Pain Scale has a sequence of numbers (from 0 to 10), where 0 represents “no pain” and 10 represents “the worst pain imaginable”. A reduction of 2 points in pain intensity is considered a minimal clinically important difference (Farrar et al. 2001). The numerical pain scale is answered by the patients at three time points: at the beginning, at the end, and 24 h after the end of each resistance training session.

### 2.12 Affect

The affective valence scale will monitor pleasure or displeasure during the three resistance training programs and will be conducted using the affective valence scale. The scale is quantified as follows: +5 to −5, corresponding, respectively, to the two antagonistic descriptors of the feeling during physical exercise and/or physical activity, which can be: “very good” and “very bad”. In addition, the affective valence scale presents other intermediate descriptors, which are: +3 = good, +1 = fairly good, 0 = neutral, −1 = fairly bad, and −3 = bad (Hardy and Rejeski 1989). The affective valence scale is completed by the patients at three times: at the beginning, at the end, and 24 h after the end of each resistance training session.

### 2.13 Subjective Perception of Effort

The intensity of each resistance training session will be quantified using the subjective perception of effort method. This is done by asking the following question: “How was your exercise session?” The answer will be given, as recommended, 30 min after the end of the session using a scale adapted from Borg (Borg 1982). Patients will be instructed to select a descriptor and then a number from 0 to 10, which may be given in decimals (e.g., 4.5). The scale used will present the following descriptors: 0 = rest; 1 = very, very easy; 2 = easy; 3 = moderate; 4 = somewhat difficult; 5 = difficult; 7 = very difficult; 10 = maximum. The maximum value (10) should be compared to the greatest physical effort experienced by the individual, and the minimum value should be compared to the state of absolute rest (0).

### 2.14 Training Volume

Training volume is one of the acute variables of resistance training that is manipulated according to the goal, level of conditioning, and specific recommendations for each individual (Pontes‐Silva, Dibai‐Filho, et al. 2023). Therefore, at the end of each training session, the total training volume of the session will be recorded in a spreadsheet. The training volume was calculated in this study as follows: number of sets × number of repetitions × load (external = kilos lifted per exercise) × subjective perception of effort (values provided by the Borg scale), which provides a value in arbitrary units (Macfarlane et al. 2017; Rausch Osthoff et al. 2018).

### 2.15 Cadence

Physical effort in resistance training can also be obtained by measuring the cadence time of each repetition during a given series (concentric phase and eccentric phase), in this case using a mathematical model that replaces the values provided by subjective effort with the effort time performed (Pontes‐Silva 2024a). Thus, in the present study, we will use a stopwatch to measure the effort time of each series, where this stopwatch will be triggered at the beginning of each movement of each series and paused at the end, with this value recorded in a spreadsheet.

### 2.16 Participant and Audience Engagement

Patients will not be involved in the design of the study, the formulation of the research question, or the development of recruitment procedures. At the end of the study, the results may be reported to the patients in the form of a presentation of the effects found on the variables studied. If superiority of one program over another is found, this will be reported to the research patients.

### 2.17 Sample Size

Sample size calculation was performed a priori using G*Power software (version 3.1.9.7) with the Chi‐squared test for comparisons between the proportions of self‐reported preferences observed in the contingency tables. We calculated the sample seeking an effect size of 0.8 in the difference in the primary outcome (preference). That is, *w* = 0.8, α = 0.05, β = 0.80, degrees of freedom = 25, non‐centrality parameter λ = 23.04, and critical Chi‐squared test = 37.65 (Gignac and Szodorai 2016; Pontes‐Silva 2023). Thus, the sample was estimated to be 36 patients.

### 2.18 Statistical Methods

For the primary outcome (preference), the number of choices of the 3 types of training will be recorded as a percentage. For the analysis of the primary outcome, we will summarize the patient's preference in a contingency table, compare the proportions using the chi‐squared test, and finally verify the effect size of the observed differences. For the secondary outcomes, statistical analyses will be performed by a blind statistician using commercial software. The independent variables of group and time (T0, T1, T2, T3, T4) will be considered for each dependent variable: pain intensity, subjective effort, affect and satisfaction. The Kolmogorov–Smirnov test was used to verify the distribution of the data and the Levene test was used to analyze the homogeneity of variance.

A mixed design analysis of variance will be conducted for the primary and secondary outcomes with a normal distribution, with time as the within‐subject factor and group as the between‐subject factor. If the data are not normally distributed, the Friedman test is used. Time × group interactions and between‐ and within‐group differences were analyzed for all variables. Finally, the Bonferroni test is used in post hoc analyses to determine whether there are differences between‐groups at different intervention times. A 5% significance level and 95% confidence interval will be used for all statistical analyses.

## 3 Results

Based on the design of this protocol, we intend to evaluate the preference of women with fibromyalgia in three different protocols in resistance training, evaluating these patients over 4 weeks where all of them will experience the three protocols. 7 days after the last week of the protocol, we will evaluate the patients' preference.

## 4 Discussion

Although resistance training is recognized as a treatment for women with fibromyalgia, it is important to note that treatment for this population in Brazil is almost exclusively pharmacological; additionally, most people who exercise do aerobic exercises (Macfarlane et al. 2017; A. M.R. de et al. 2020). Therefore, patients with fibromyalgia in Brazil undergoing pharmacological and non‐pharmacological treatment do not receive complete treatment, as resistance training is not included (A. M.R. de et al. 2020).

The benefits of resistance training for patients with fibromyalgia are indisputable, such as: improvement in pain, function, quality of life, pain points, and improvement in physical fitness (Astley et al. 2021). However, we still observe a high abandonment of activities by these patients; some say that part of this situation is because of previous bad experiences with physical exercise and the beliefs of the patients (Russell et al. 2018).

It is plausible to assume that low adherence or abandonment of any type of treatment, in this case physical exercise, is not due to just one factor exclusively. It is very likely that this situation is materialized by multiple factors, such as: lack of patient education (Souza et al. 2008), lack of specialized care (Barton et al. 2021), lack of financial resources and educational background of the patient (A. T. R.C. de et al. 2017), the lack of willingness of the health professional to raise awareness among the patient and poor communication between the health professional and the patient can influence the prognosis and success of the treatment (Georgopoulou et al. 2018).

In contrast, although we recognize the complexity of non‐adherence and the real danger of a sedentary lifestyle for rheumatic patients regardless of age, we have observed an increasing number of studies in the literature that further reinforce the benefits of resistance training for patients with fibromyalgia (Rodríguez‐Domínguez et al. 2024), studies that investigate one of the acute variables of training, intensity (Rodríguez‐Almagro et al. 2023), and we have even observed studies that question modalities that were previously considered beneficial for patients with fibromyalgia (Pontes‐Silva, Dibai‐Filho, et al. 2023).

Importantly, distinguishing between prescribed and preferred exercise intensity can significantly impact the outcomes of resistance training for patients with fibromyalgia; da et al. (2018) demonstrated that patients reported lower pain perception during and after resistance exercise sessions performed at a preferred intensity compared to a prescribed intensity. Despite the fact that both intensities were matched in volume, the preferred intensity condition was associated with greater potential for adherence and more favorable affective responses. These findings underscore the importance of including resistance training in treatment plans and tailoring its variables, such as intensity, to patient preferences. This approach aims to minimize symptom exacerbation and increase adherence.

However, there are few studies that take into account the preference of the type of physical activity preferred by healthy individuals and especially by people with fibromyalgia (Pontes‐Silva 2024b). In addition, studies that have acknowledged the importance of patient preference in choosing exercise have shown superior outcomes in terms of adherence, symptom improvement, and physical fitness (Pontes‐Silva, Nunes, et al. 2023).

In addition to its physical benefits, considering exercise preference has been shown to improve psychological outcomes in individuals with fibromyalgia. For example, Andrade et al. (2020) found that patients who engaged in their preferred form of exercise reported better mental health indicators, including reduced symptoms of anxiety and depression, compared with patients who performed non‐preferred activities. These findings highlight the biopsychosocial nature of fibromyalgia and the importance of incorporating patient‐centered approaches into treatment planning. Allowing patients to choose exercise modalities or characteristics that align with their preferences may lead to more comprehensive, sustainable, and psychologically supportive interventions.

Therefore, in addition to the aforementioned reasons for the importance of incorporating patient preference into treatment planning, we also reiterate the pillars of evidence‐based practice, emphasizing that patient preference is part of the triad of this relevant behavior in clinical decision‐making by all health professionals (Garber et al. 2011).

One of the main strengths of this study is its focus on patient preference in relation to resistance training volume, a variable often overlooked in intervention designs. By directly evaluating this component, our protocol adheres to the clinical relevance of individualized treatment and the principles of evidence‐based practice. Furthermore, using three distinct resistance training volumes provides a comprehensive framework for detecting nuanced differences in patient preferences. This approach may enhance the ecological validity and applicability of our findings in real‐world clinical settings.

Another important strength of this research is its contextualization within the Brazilian healthcare system, where fibromyalgia treatment is predominantly pharmacological and access to comprehensive non‐pharmacological care is limited. By addressing these systemic challenges and proposing a low‐cost, scalable intervention, such as resistance training, our study offers a practical, culturally relevant contribution that could influence future policies and professional practices in other low‐ and middle‐income countries.

Nevertheless, some limitations should be considered. Since this is a protocol, the actual behavior of the patients, such as their adherence and preferences, will only be fully understood upon implementation. Additionally, our study is restricted to women, which may limit the generalizability of the findings to men with fibromyalgia. Additionally, while we examined an important acute variable, training volume, we standardized other parameters, such as intensity, frequency, and exercise modality, which could influence patient experience and perceived preference.

Despite its limitations, this study is novel in its patient‐centered approach and specific focus on training volume preference, an understudied aspect of fibromyalgia management. While the literature extensively covers the effectiveness and safety of exercise interventions, few studies have investigated which training characteristic patients prefer. Our clinical trial protocol fills this gap by providing new insights that may help design more acceptable, sustainable, and individualized exercise programs for women with fibromyalgia. It also serves as a basis for future studies in rheumatology and exercise science.

## 5 Implications of Physiotherapy Practice

- This protocol will support the first trial examining the preference of women with fibromyalgia for different volumes of resistance training.
- We will check whether exercise volume preference is related to subjective perception of exertion in women with fibromyalgia.
- We will check whether exercise volume preference is *related* to pain intensity in women with fibromyalgia.

## Author Contributions

M.C.S. and B.H.C.: conceptualization. B.H.C.: data curation, investigation, and statistical analysis. All authors: methodology, validation, visualization, writing – original draft, writing – review and editing.

## Ethics Statement

This study was approved by the Research Ethics Committee of the Universidade Federal do Rio Grande do Norte (Report number: 6.303.419; October 20, 2024).

## Consent

Informed consent was obtained from all subjects and/or their legal guardian(s). All respondents participated in this study freely and with their consent. All experiments were conducted in accordance with the tenets of the Declaration of Helsinki.

## Conflicts of Interest

The authors declare no conflicts of interest.

## Data Availability

The data and materials in this paper are available from the corresponding author on request.
